# Comparison of analysis methods to classify cholera hotspots in Ethiopia from 2015 to 2021

**DOI:** 10.1038/s41598-024-56299-5

**Published:** 2024-04-03

**Authors:** Yeshambel Worku Demlie, Sandra Moore, Jessica Dunoyer, Dereje Muluneh, Mukemil Hussen, Mesfin Wossen, Moti Edosa, Bertrand Sudre

**Affiliations:** 1 Public Health Emergency Management, Ethiopian Public Health Institute (EPHI), Addis Ababa, Ethiopia; 2 Prospective and Cooperation, 1 place Gabriel Péri, Vieux port, 13001 Marseille, France; 3 Health Section, UNICEF Ethiopia, UNECA Compound, Zambezi Building, Box 1169, Addis Ababa, Ethiopia

**Keywords:** Ethiopia, *Vibrio cholerae*, Cholera, Cholera dynamics, Cholera hotspots, Cholera elimination, Targeted interventions, Preparedness, Prevention, Bacterial infection, Epidemiology

## Abstract

Cholera continues to represent a major public health concern in Ethiopia. The country has developed a Multi-sectoral National Cholera Elimination Plan in 2022, which targets prevention and control interventions in cholera hotspots. Multiple methods to classify cholera hotspots have been used in several countries. Since 2014, a classification method developed by United Nations Children's Fund has been applied to guide water, sanitation and hygiene interventions throughout Sub-Saharan Africa based on three outbreak parameters: frequency, duration and standardized attack rate. In 2019, the Global Task Force on Cholera Control (GTFCC) proposed a method based on two parameters: average annual cholera incidence and persistence. In 2023, an updated GTFCC method for multisectoral interventions considers three epidemiological indicators (cumulative incidence, cumulative mortality and persistence,) and a cholera-case confirmation indicator. The current study aimed to classify cholera hotspots in Ethiopia at the woreda level (equivalent to district level) applying the three methods and comparing the results to optimize the hotspot targeting strategy. From 2015 to 2021, cholera hotspots were located along major routes between Addis Ababa and woredas adjacent to the Kenya and Somalia borders, throughout Tigray Region, around Lake Tana, and in Afar Region. The multi-method comparison enables decision makers to prioritize interventions according to a sub-classification of the highest-priority areas.

## Introduction

Cholera is caused by toxigenic forms of the bacterium *Vibrio cholerae* O1 and O139, which is primarily contracted by ingesting contaminated water or food^1^. Cholera outbreak dynamics are influenced by various risk factors such as limited access to basic water supply, sanitation infrastructure, hygiene facilities (WASH), which renders the population vulnerable to disease transmission^2^. Secondary factors such as severe climate events (e.g. droughts and flooding), conflict and population displacement can exacerbate outbreaks, impede access to healthcare facilities, and hinder response efforts^3–5^. As a result, cholera outbreak frequency, duration and incidence may vary depending on the location, context, and the population affected.

Cholera continues to represent a major public health concern in Ethiopia. Between 2019 and 2021, Ethiopia reported a total of 15,515 suspected cholera cases^6^. In response to this persistent public health threat, the Ethiopian Public Health Institute (EPHI) has recently developed an evidence-based Multi-sectoral National Cholera Elimination Plan^7^. The strategy aims to interrupt cholera transmission in the country by identifying cholera hotspots at the woreda level (equivalent to district level) and improving access to WASH services in high-risk kebeles within these hotspots^7^.

A cholera hotspot is a “geographically limited area where environmental, cultural and/or socioeconomic conditions facilitate the transmission of the disease, and where cholera persists or reappears regularly”^8^. As these areas play a central role in the spread of cholera outbreaks, multisectoral interventions should target these areas to prevent and control cholera outbreaks, prioritizing the most at-risk hotspots to most efficiently use limited resources. Several hotspot classification methods have been implemented in Africa over the past decade. The first method was developed by the United Nations Children's Fund (UNICEF) West and Central Africa Regional Office (WCARO) in 2014 based on the analysis of outbreak frequency, outbreak duration and standardized outbreak attack rate per cholera surveillance unit (CSU) (ideally at the district level)^9^. Based on these three parameters, CSUs are then classified into four priority categories for targeted interventions. This approach was first applied using long time-series in 12 countries in West Africa in 2014, which was then updated and applied on 14 countries in 2018^9^ and eight countries in East and Southern Africa in 2017–2018^10^. A second method was proposed by the Global Task Force on Cholera Control (GTFCC) in 2019, which uses two yearly surveillance parameters: mean annual incidence of suspected cholera cases and cholera persistence (the number of weeks per year with at least one suspected case reported). With the GTFCC 2019 method, CSUs are classified into three priority categories (high-, medium- and low-priority)^8^. The core principle of this method has been used in various countries; in some cases, additional parameters have been included, such as WASH indicators in Kenya or case fatality ratio in Ethiopia^7,11,12^. In 2023, an updated method was proposed by the GTFCC to identify priority areas for multisectoral interventions (PAMIs) in countries with moderate to high cholera transmission. With this method, priority areas are identified based on three epidemiological parameters (cumulative incidence, cumulative mortality and cholera persistence, as defined above) and a cholera test positivity indicator^13^. In addition to these three main methods, other country-specific ad-hoc classification methods have been implemented using a combination of different epidemiological indicators and contextual cholera risk factors^14–16^. To date, no study has compared the three main cholera hotspot classification methods to adapt targeting strategies.

The current study aimed to identify and classify cholera hotspots in Ethiopia at the woreda level applying the three main classification methods. The results of the three methods were compared, highlighting the pros and cons of each approach.

## Results

**Figure 1:**
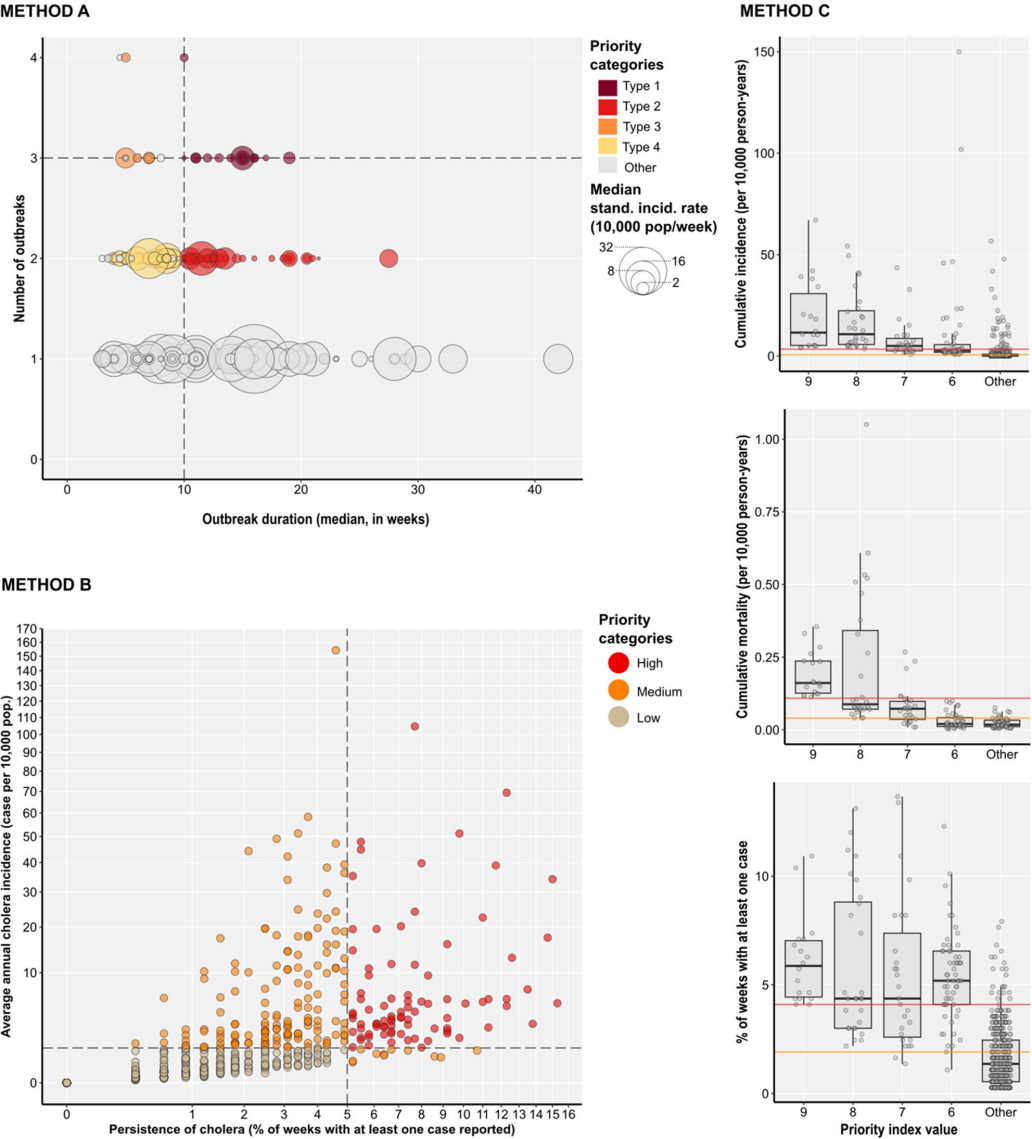
Cholera hotspots plotted according to the epidemiological parameters for each classification method. Method (**A**) Hotspots are color-coded according to the four classification types plotted against the three classification parameters [X-axis: outbreak duration (median in weeks), Y-axis: the number of outbreaks, and circle symbol area: median standardized incidence rate (10,000 person-weeks), horizontal dashed line: threshold at P95, vertical dashed line: threshold at P60]. Method (**B**) Hotspots are color-coded according to the three priority categories plotted based on the two classification parameters [X-axis: persistence (% of weeks with at least one case reported over the study period), Y-axis: average of the yearly incidence (per 10,000 pop.), horizontal dashed line: threshold at 1 case per 10,000 pop., vertical dashed line: threshold at 5%]. Method (**C**) Hotspots are distributed according to the three classification parameters and the six upper priority index values (lower values zero, two, three, four and five are regrouped under “Other”). Horizontal lines correspond to thresholds for each indicator at the median value (in orange) and at P80 (in red).

From week 37 2015 to week 52 2021, cholera hotspots in Ethiopia, at the woreda level, were classified using three analysis methods (Fig. 1).

**Figure 2:**
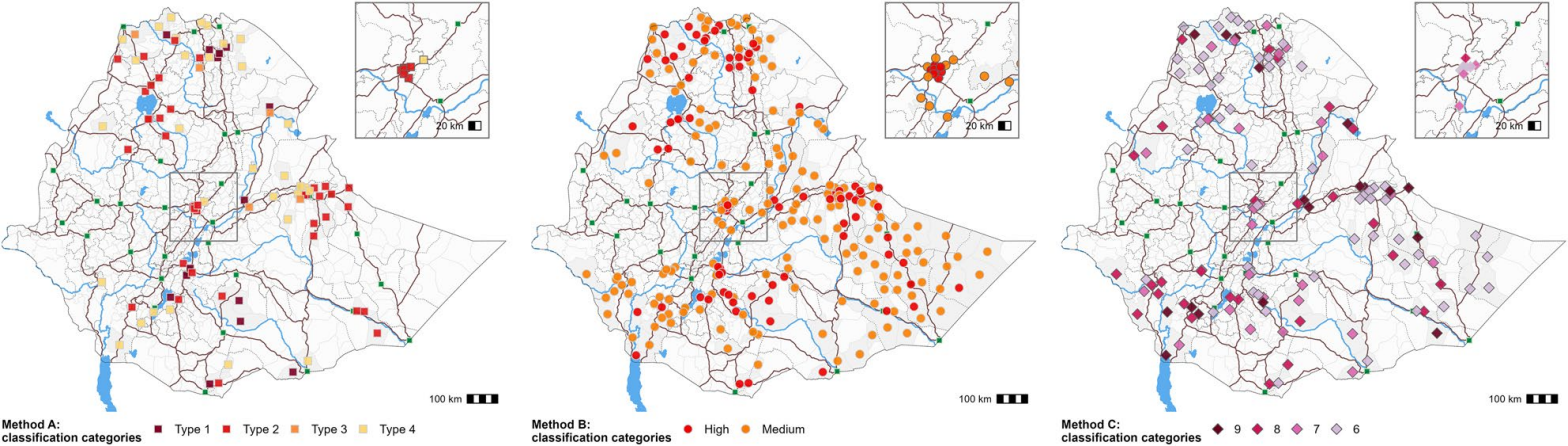
Map of cholera hotspots for each classification method. Dark brown lines correspond to roads, green squares correspond to main urban centers, and blue lines and areas correspond to waterbodies. Gray areas correspond to each woreda classified as a hotspot. The maps were generated using the software QGIS V3.28 Firenze^33^ and R-4.3.0^32^ (with ggmap package).

Using Method A, 90 CSUs were classified as cholera hotspots (Types 1–4) over the course of the study period. A total of 54 CSUs were classified as Type 1 or Type 2 hotspots (Table 1). In terms of outbreak duration, Type 1 and 2 hotspots had a median outbreak duration ≥ 10.5 weeks, with maximum outbreak durations of 19 weeks and 27.5 weeks, respectively. In terms of outbreak frequency, 19 CSUs notified ≥ three outbreaks, of which 14 were classified as Type 1 and five were classified as Type 3 (Table 1; Fig. 1, Method A). Type 1 to 4 hotspots were located in Tigray Region (notably around Mekelle), along the borders with Sudan and Eritrea, in northern Amhara Region (around Lake Tana), in Afar Region along the border with Djibouti, in Addis Ababa (the capital city), along main roads toward the east (connecting Addis Ababa with Dire Dawa and Jigjiga), and along roads to Kenya (Shashemene, Hawassa and Arba Minch) and Somalia. The majority of Type 1 hotspots were located in Tigray Region, along routes connecting Addis Ababa with Kenya and Somalia, Bale Zone in Oromia Region, and along the Kenya and Somalia borders (Moyale and Dolo Odo, respectively) (Fig. 2).

**Table 1:** Epidemiological characteristics of the cholera hotspots identified using methods A, B and C.

	CSUs		Population			Cases			Deaths			Num. of CSUs stratified by outbreak number				Num. of outbreaks	Persistence			Average cum. incidence	Average cum. mortality
	N	Rel. %	N	Rel. %	Cum. %	N	Rel. %	Cum. %	N	Rel. %	Cum. %	1	2	3	4	Total	Sum of weeks with ≥ 1 case	Rel. %	Average (weeks)	(per 10,000 person-years)	(per 10,000 person-years)
Category																					
Method A																					
Type 1	14	1.4	2,028,200	2.1	2.1	8361	8.4	8.4	55	8.6	8.6	0	0	13	1	43	523	9.6	37.4	10	0.1
Type 2	40	3.9	7,338,200	7.6	9.7	22,486	22.6	31	115	18.1	26.7	0	40	0	0	80	1002	18.4	25	6.6	0
Type 3	5	0.5	340,400	0.4	10.1	1272	1.3	32.3	22	3.5	30.2	0	0	4	1	16	99	1.8	19.8	6.4	0.1
Type 4	31	3	2,353,900	2.5	12.6	7483	7.5	39.8	105	16.5	46.7	0	31	0	0	62	410	7.5	13.2	6.9	0.1
Other	943	91.3	83,945,200	87.4	100	59,946	60.2	100	340	53.4	100	256	19	6	2	320	3424	62.7	3.6	1.8	0
Subtotal	1033	100.1	96,005,900	100		99,548	100		637	100		256	90	23	4	521	5458	100			
Method B																					
High	86	8.3	11,882,200	12.4	12.4	52,460	52.7	52.7	247	38.8	38.8	22	43	19	2	173	2206	40.4	25.7	10.2	0
Medium	164	15.9	11,995,200	12.5	24.9	41,669	41.9	94.6	343	53.8	92.6	116	36	4	2	208	1751	32.1	10.7	8.7	0.1
Low	783	75.8	72,128,500	75.1	100	5419	5.4	100	47	7.4	100	118	11	0	0	140	1501	27.5	1.9	0.1	0
Subtotal	1033	100	96,005,900	100		99,548	100		637	100		256	90	23	4	521	5458	100			
Method C																					
9	18	1.7	1,021,500	1.1	1.1	12,510	12.6	12.6	132	20.7	20.7	6	7	4	1	36	403	7.4	22.4	19.6	0.2
8	28	2.7	2,126,300	2.2	3.3	18,839	18.9	31.5	221	34.7	55.4	13	9	6	0	49	602	11	21.5	16.9	0.2
7	27	2.6	3,163,200	3.3	6.6	15,425	15.5	47	121	19	74.4	13	8	5	1	48	535	9.8	19.8	8	0.1
6	60	5.8	7,870,300	8.2	14.8	25,735	25.9	72.9	85	13.3	87.7	21	34	4	1	105	1184	21.7	19.7	10	0
5	66	6.4	6,444,600	6.7	21.5	17,327	17.4	90.3	41	6.4	94.1	44	17	4	1	94	862	15.8	13.1	8.1	0
4	68	6.6	6,466,000	6.7	28.2	5242	5.3	95.6	22	3.5	97.6	58	9	0	0	76	620	11.4	9.1	1.8	0
3	96	9.3	9,694,500	10.1	38.3	2842	2.9	98.5	15	2.4	100	61	6	0	0	73	658	12.1	6.9	0.7	0
2	207	20	21,856,300	22.8	61.1	1628	1.6	100	0	0	100	40	0	0	0	40	594	10.9	2.9	0.1	0
0	463	44.8	37,363,300	38.9	100	0	0	100	0	0	100	0	0	0	0	0	0	0	0	0	0
Subtotal	1033	99.9	96,006,000	100		99,548	100		637	100		256	90	23	4	521	5458	100			

*Rel*. % relative percentage, *Cum*. % cumulative percentage.

Using Method B, 86 CSUs were classified as high priority hotspots, 164 CSUs were classified as medium priority hotspots, and the remaining 783 CSUs were classified as low priority hotspots. For the high-priority hotspots, the mean incidence broadly ranged from 1.02 to 105, with an average of approximately 10 cases per 10,000 population and an average cholera persistence of 26 weeks. The medium-priority CSUs can be split in two sub-groups: (1) 11 hotspots with low incidence and high persistence and (2) 153 hotspots with high incidence and low persistence (Table 1; Fig. 1). Method B hotspots were widespread throughout the country. Hotspots with high incidence were located in northern Afar Region, Somali Region, Southern Nations, Nationalities, and Peoples' (SNNP) Region and South West Ethiopia Peoples' (SWEP) Region. Hotspots with high persistence were located in Somali Region, Dire Dawa Region, Harari Region, Oromia Region (including Bale Zone), northern Afar and Tigray Region. This method classified areas affected by a single epidemic as medium-priority cholera hotspots (in Somali Region and SNNP in 2017 as well as SWEP Region in 2021 and 2021) (Fig. 2).

For Method C, the median priority index of six was defined as threshold to define priority areas. A total of 133 hotspots had a priority index ≥ six, which corresponds to an estimated population of 7.8 million (approx. 15% of the total population), 72.2% of all cases and 87.7% of deaths (Table 1; Fig. 1). Method C hotspots were distributed in a similar pattern to that of Method B, although fewer CSUs were identified as hotspots using Method C. Several high-priority hotspots (priority index ≥ 8) were located in Tigray Region, SNNP Region, SWEP Region, along the eastward road from Addis Ababa, and Somali Region (Fig. 2). High-priority hotspots in Somali Region included urban areas with high cumulative incidence and very high persistence due to the epidemic in 2017. Hotspots with very high morality were essentially located along the border with South Sudan (SWEP and Gambela Regions). Maps of the parameters used in each method are available in the Supplementary Materials 1, 2 and 3.

**Figure 3:**
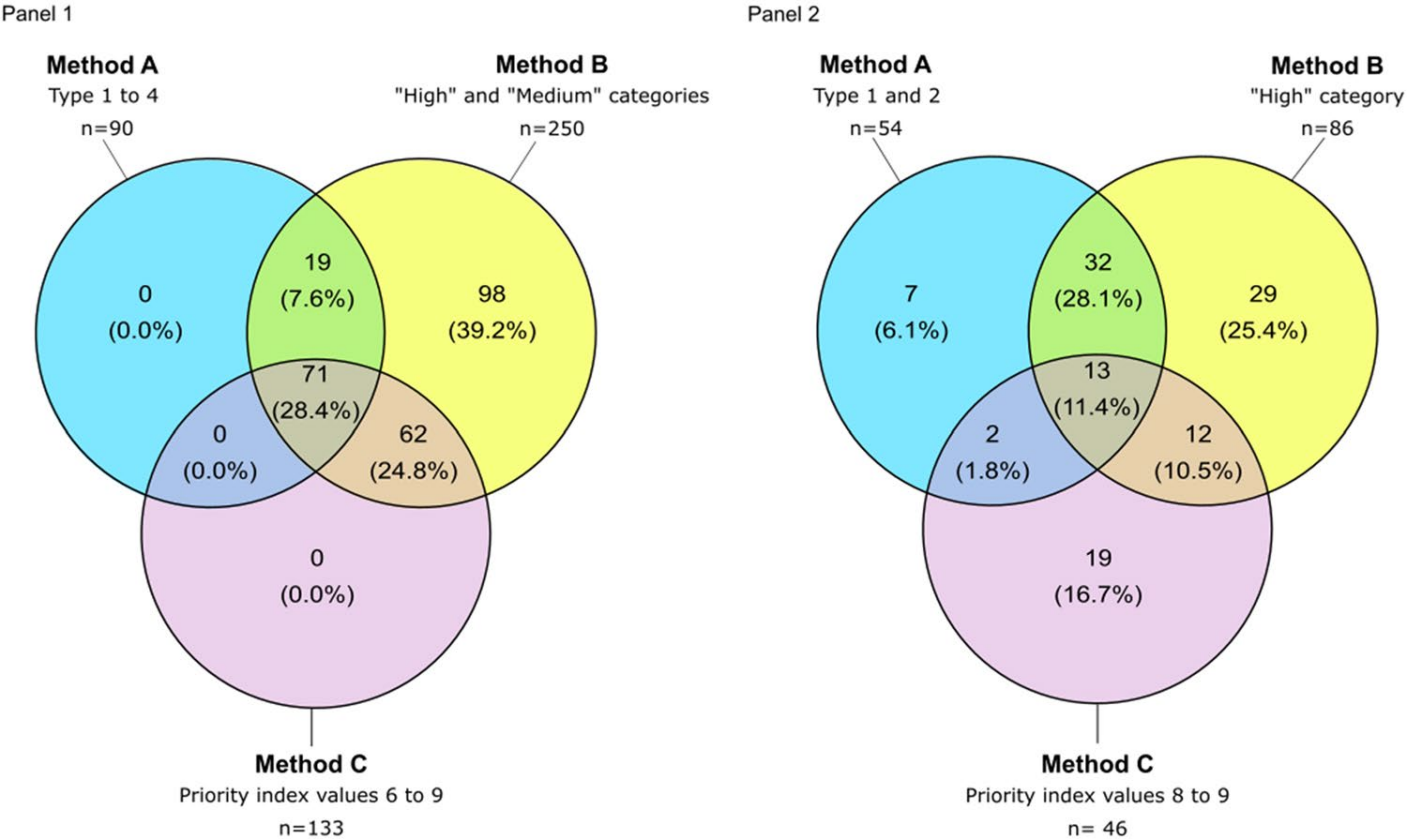
Venn diagram for each cholera hotspot classification method. The Venn diagram shows the logical relationships between the method A, B and C results. Circles that overlap have common hotspots (number of CSUs and percentage of the overall total is provided). Areas that do not overlap represent hotspots identified by only one method. Panel (**1**) The relationship between all 250 hotspots across the three classification methods. Panel (**2**) The relationship between a subset of only high-priority hotspots for each classification method (n = 112) as follows: Method A (Types 1 and 2), Method B (High-priority) and Method C (priority indexes 8 and 9).

**Figure 4:**
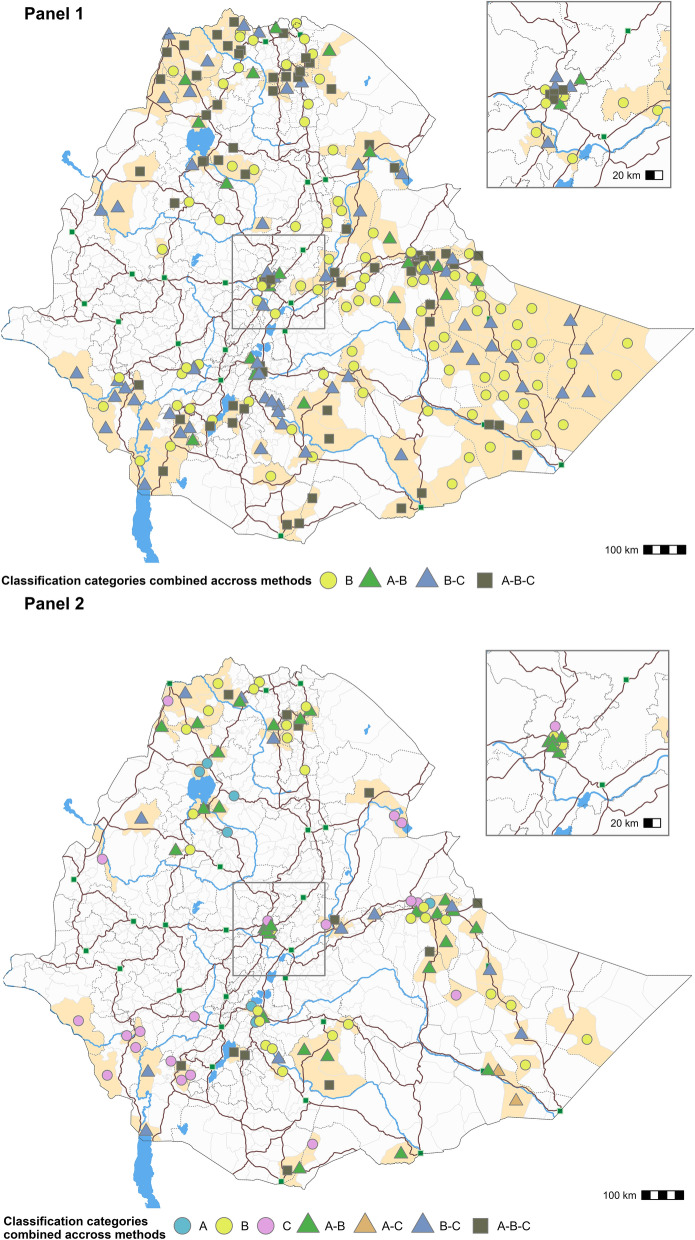
Localization of each group from the Venn diagram for all cholera hotspots (Panel (**1**)) and high-priority cholera hotspots (Panel (**2**)). Legend: Dark brown lines correspond to roads, green squares correspond to main urban centers, and blue lines and areas correspond to waterbodies. Light brown areas correspond to the CSUs. The maps were generated using the software QGIS V3.28 Firenze^33^ and R-4.3.0^32^ (with ggmap package).

The relationship between the results of the three methods are illustrated in Fig. 3, and the epidemiological features for each comparison are provided in Table 2. When comparing all hotspots identified by the three methods, all hotspots identified by Method A were also identified by Method B (Fig. 3, Panel 1). Likewise, each hotspot identified by Method C was also identified by Method B (Fig. 3, Panel 1). Most of the hotspots identified only by Method B were located in remote areas (especially in Somali Region), whereas hotspots identified by all three methods were located along major roads, around major waterbodies or along international borders. The majority of hotspots in Somali Region were not identified by Method A as many of these CSUs were only affected by a single outbreak (Fig. 4, Panel 1).

**Table 2:** Epidemiological characteristics for each component of the Venn diagram (Panels 1 and 2).

	CSUs		Population		Cases		Deaths		Num. of CSUs stratified by outbreak number				Num. of outbreaks	Persistence			Average incidence	Average mortality
	N	Rel. %	N	Rel. %	N	Rel. %	N	Rel. %	1	2	3	4	N	Sum of weeks with ≥ 1 case	Rel. %	Average (weeks)	(per 10,000 person-years)	(per 10,000 person-years)
Method combination																		
Set diagram: panel 1																		
A-B	19	1.8	2,603,700	2.7	3481	3.5	10	1.6	0	17	2	0	40	309	5.7	16.3	2.7	0
A-B-C	71	6.9	9,457,000	9.9	36,121	36.3	287	45.1	0	54	15	2	161	1725	31.6	24.3	8.4	0.1
B	98	9.5	7,092,400	7.4	18,139	18.2	21	3.3	85	4	2	1	103	924	16.9	9.4	6.2	0
B-C	62	6	4,724,200	4.9	36,388	36.6	272	42.7	53	4	4	1	77	999	18.3	16.1	16.9	0.1
Other	783	75.8	72,128,500	75.1	5419	5.4	47	7.4	118	11	0	0	140	1501	27.5	1.9	0.1	0
Subtotal	1033	100	96,005,800	100	99,548	100	637	100	256	90	23	4	521	5458	100			
Set diagram: panel 2																		
A	7	0.7	1,663,400	1.7	1047	1.1	13	2	0	6	1	0	15	163	3	23.3	1.1	0
A-B	32	3.1	6,259,200	6.5	19,878	20	44	6.9	0	27	4	1	70	884	16.2	27.6	5.6	0
A-B-C	13	1.3	1,328,000	1.4	8198	8.2	90	14.1	0	5	8	0	34	446	8.2	34.3	12.6	0.1
A-C	2	0.2	115,800	0.1	1724	1.7	23	3.6	0	2	0	0	4	32	0.6	16	26.4	0.2
B	29	2.8	3,380,900	3.5	12,094	12.1	33	5.2	16	8	5	0	47	595	10.9	20.5	9.6	0
B-C	12	1.2	914,000	1	12,290	12.3	80	12.6	6	3	2	1	22	281	5.1	23.4	21.2	0.1
C	19	1.8	789,900	0.8	9137	9.2	160	25.1	13	6	0	0	25	246	4.5	12.9	18.6	0.3
Other	919	89	81,554,700	84.9	35,180	35.3	194	30.5	221	33	3	2	304	2811	51.5	3.1	1.2	0
Subtotal	1033	100	96,005,900	99.9	99,548	100	637	100	256	90	23	4	521	5458	100			

*Rel*. % relative percentage, *Cum*. % cumulative percentage.

When comparing only high-priority hotspots identified by each method (a total of 112 hotspots), 11.4% (13 hotspots) were identified by all three methods, 28.1% (32 hotspots) were only identified by Methods A and B, 10.5% (12 hotspots) were only identified by Methods B and C, and 2% were only identified by Methods A and C. However, 48.2% of these high-priority hotspots were identified only by a single method, notably using Method B (19 hotspots) and Method C (29 hotspots) (Fig. 3, Panel 2). All seven high-priority hotspots uniquely identified with Method A were affected by multiple outbreaks, while 16 high-priority hotspots identified uniquely with Method B were only affected by a single outbreak. Likewise, high-priority hotspots solely identified using Method C experienced either a single outbreak (13 CSUs) or two outbreaks (six CSUs) over the course of the entire study period (Table 2). High-priority hotspots identified by all three methods represent 9.6% of all cases and 14.1% of all deaths; these hotspots were all affected by multiple outbreaks. These hotspots were located in border areas (e.g. Moyale, etc.), along major roads, near large waterbodies, and in urban areas (Fig. 4, Panel 1). Many high-priority hotspots only identified by Method C were located in the southwest, (SNNP and SWEP Regions) (Fig. 4, Panel 1), where a cholera epidemic spread in previously unaffected remote areas causing significant mortality (Supplementary Material 3).

## Discussion

In this comprehensive study, three analysis methods were applied to identify and classify cholera hotspots in Ethiopia, at the woreda level, from September 2015 to December 2021. Overall, high-priority cholera hotspots were mainly located along major routes between Addis Ababa and the Kenya and Somalia borders, throughout Tigray Region, around Lake Tana (in Amhara Region), and in Afar Region along the Ethiopia-Djibouti road. The results of the classification methods were then compared to identify the best approach for Ethiopia to implement targeted strategies to achieve the objective of cholera elimination.

Classification methods A, B and C identified a total of 90, 250 and 133 cholera hotspots, respectively. A total 71 CSUs were identified as hotspots by all three methods. Assessing only the high-priority hotspots (Types 1–2, Method A; high-priority, Method B; and priority index ≥ 8, Method C), a total of 54, 86 and 46 hotspots were identified by each method, respectively, among which only 13 hotspots were identified by all three methods.

Over the course of the study period, multiple regions across Ethiopia were vulnerable to cholera outbreaks due to a variety of factors including poor access to water and sanitation^17^, severe weather (e.g. drought and flooding)^18,19^ and cross-border transmission with neighboring Somalia and Kenya^20^. However, due to constrained resources, cholera elimination strategies must prioritize prevention efforts targeting a restricted sub-set of cholera hotspots. A cholera hotspot is defined an area “*where cholera persists or reappears regularly*” and thus plays a critical role in outbreak diffusion to unaffected areas.

In line with the cholera hotspot definition, Method A classifies hotspots based on both outbreak duration and outbreak frequency. As a result, woredas affected by a single outbreak over the course of the entire study period were not identified as hotspots by Method A, although many of these woredas were classified by Methods B and C. Method A also resulted in a more restricted list of overall hotspots, with four priority sub-groups according to the distinct outbreak dynamics, thus enabling limited resources to be targeted and prioritized according to cholera transmission dynamics. Method A was initially developed to target areas with frequent outbreaks for long-term WASH interventions.

Classification Method B was based solely on cholera persistence and incidence. Due to the widespread nature of cholera epidemics in Ethiopia over the past six years, this approach identified nearly 25% of all woredas in the country as cholera hotspots (250 hotspots). However, targeting such a large number of hotspots would likely challenge the implementation of long-term water and sanitation infrastructure investments, especially with competing public health priorities. To improve this method, the GTFCC updated the classification approach in 2023 (Method C).

Compared with Method B, Method C identified a more restricted set of hotspots affected by recurrent outbreaks and/or outbreaks of long duration with substantial transmission intensity. This method also includes a mortality indicator to account for the objectives of the *Global Roadmap to 2030* to reduce cholera deaths by 90%^13,21^. However, this indicator is primally based on surveillance data of deaths recorded in healthcare facilities and would overlook community deaths in areas with limited healthcare facility coverage. For this study, the data provided for Somali Region in 2017 did not include cholera-related deaths. In this remote and rural area with a high proportion of pastoralist populations, the community case and death numbers are also likely underestimated^22^. Due to these surveillance limitations and missing data for Somali Region, Method C alone is likely not currently adapted to the Ethiopia context.

Regardless of the classification method(s) applied, the hotspot classification should be interpreted, adjusted and validated by WASH specialists and public health experts that understand the cholera risk factors in the country^13^. Key areas that play an important role in cholera dynamics may not be prioritized by a given method, which may be due missing surveillance data, etc. A validation workshop provides the opportunity to agree on the final hotspot list based on the analysis and manually adjust the classification according to the specific context as needed. This process is also critical to ensure ownership by the public health authorities and other actors involved in cholera control. Furthermore, the most appropriate method or a multi-method approach should be selected and adapted depending on the parameters included in the analysis, data available for the analysis, and the country-specific conditions.

Some study limitations should be noted. The three methods were applied based on the recommended thresholds; however, these thresholds should ideally be set by a panel of experts during a validation workshop. Suspected cholera cases have not been reported from Tigray Region since 2019. Given the insecurity context in the region in 2020 and 2021, the cholera burden may be underestimated due to challenges in healthcare access and disease surveillance limitations. Nevertheless, it is unlikely that a major cholera outbreak in Tigray Region would spread undetected. Regarding the epidemic in Somali Region in 2017, the aggregated databases provided for the analysis did not include cholera-related deaths; as a result, the deaths in this region were significantly underestimated. The total annual cholera case data by region from the WHO Ethiopian country office used to perform the gap analysis were only available for the period 2015–2018; nevertheless, the gap analysis for the years 2019 and 2021 was conducted using the annual cholera case numbers from the WHO Weekly epidemiological record data. As WASH indicator data at the woreda level was unavailable, we were unable to assess the WASH profile of each hotspot type.

These results highlight several actions to further strengthen cholera elimination efforts in Ethiopia. As cholera hotspot patterns can be dynamic due to various factors such as population movement, socio-economic variables and climate factors, the hotspot analysis should be regularly updated. Indeed, a parallel study conducted by Moore et al.^23^ provides a detailed description of the dynamic spatiotemporal characteristics of cholera epidemics in Ethiopia during the same time period. The classification exercise should also be conducted ad hoc if the cholera context in the country evolves or major events occur that may drive cholera transmission (e.g. extreme weather events, conflicts). Additional classification exercises could also test the predictive power of each method applied and monitor cholera elimination progress. To establish a comprehensive understanding of the disease dynamics in each woreda, additional data on underlying factors that contribute to disease transmission should be included in the hotspot classification analysis. Incorporating WASH data into this analytical framework can help to identify high-risk areas where inadequate water and sanitation infrastructures and hygiene practices contribute to cholera transmission, while vaccination data can help to prioritize areas where targeted vaccination is required to maintain herd immunity. Finally, supplementary studies should be performed, especially in high-priority hotspots, to better identify populations regularly affected by cholera and contextual factors driving cholera transmission. In urban areas, this level of analysis is instrumental to target preparedness and prevention interventions to the most relevant populations.

This study sheds light on three complementary methods to classify cholera hotspots, a pivotal step in developing a National Cholera Plan. Comparing the results of each method by analyzing cholera data from Ethiopia for the 2015–2021 period, we have gained a comprehensive understanding of the strengths and limitations of the distinct approaches. These results underscore the importance of a multifaceted approach to cholera hotspot classification. The type of method applied should be context-specific, taking into consideration factors such as data availability, data analysis resources and capacity, and the distinct epidemiological landscape. To inform more effective strategies to identify and classify cholera hotspots going forward, additional efforts should aim to identify country-specific factors that influence cholera dynamics in major hotspot areas and adapt the analysis method accordingly. Regardless of the method(s) applied, it is important to allow for subsequent manual adjustment of the final hotspot ranking during a validation workshop with country stakeholders, thereby enabling the flexibility of a tailored strategy that harnesses the strengths of the method(s) to ultimately enhance cholera elimination efforts. Furthermore, it is critical to identify and detail key interventions per pillar and per hotspot type. Overall, these results provide valuable insights for public health policymakers to prepare for and prevent further outbreaks in a targeted manner, ultimately saving lives in vulnerable communities across Ethiopia and beyond.

## Methods

### Study design and site

In this retrospective cross-sectional study, we used cholera data from Ethiopia from week 37 2015 to week 52 2021 to apply three different methods to classify cholera hotspots.

Ethiopia is located in the Horn of Africa. According to the 2021 administrative divisions, Ethiopia comprises 13 regional states, 92 zones and 1040 woredas (equivalent to districts). The woredas are further divided into kebeles. The estimated 2021 population of Ethiopia is 103,610,998 inhabitants^24^. The most populated city and national capital is Addis Ababa, which hosts an estimated 3,780,000 people (approximately 4% of the country’s population)^24^. Ethiopia is a landlocked country with a vast highland complex of mountains and plateaus divided by the Great Rift Valley, which runs southwest to northeast and is surrounded by lowlands, steppes or deserts^25^.

### Cholera case definition

A suspected cholera case is defined as an individual with one of the two conditions^26^:

- A patient aged 5 years or more who develops severe dehydration or dies from AWD, in an area where the disease is not known to be present.
- Any patient who develops AWD, with or without vomiting, in an area where there is a cholera epidemic.

Furthermore, in the health post and community levels, a suspected cholera case (often referred to as the community case definition) can be defined as follows: any person five years of age or more with profuse AWD and/or vomiting^26^.

A confirmed cholera case refers to a suspected case in which *Vibrio cholerae* O1 or O139 has been isolated from stool via culture.

### Cholera data sources

Four sources of cholera data were available for the epidemiological analysis: regional line lists, regional aggregated databases (daily), and data templates.

WHO databases (total cases per woreda) and WHO Weekly epidemiological record data were used to identify data gaps in the EPHI databases initially provided. Any missing data was subsequently requested from the EPHI.

#### Line lists

Line lists of suspected cholera cases and deaths for the period week 37 2015 to week 52 2021 were provided by the Disease and Health Event Surveillance and Response Department at the EPHI.

#### Aggregated databases

Aggregated databases were used to supplement data gaps in the line list data. The daily aggregated data (per woreda) was converted into a weekly database for the analysis. The aggregated databases (region and year) were as follows: Somali 2017 (weeks 1–37), Dire Dawa (2017), SNNP (Southern Nations, Nationalities, and Peoples' Region) (2017), SNNP (2020), Benishangul Gumz (2017) and Sidama (2020).

#### Data templates

Two types of data templates were completed by the regions to supplement remaining data gaps: (1) aggregated total cases and deaths per woreda and (2) outbreak start and end date, total cases and deaths per woreda.

#### Annual totals for gap analysis

For the period 2015–2018, the total annual cholera cases by region were obtained from the WHO Ethiopian country office. For the years 2019 and 2021, the annual cholera case numbers were obtained from the WHO Weekly epidemiological record^27–29^. The total annual cholera cases by region for the period 2019–2021 were unavailable.

### Population data

The Ethiopian population data projections per woreda for the year 2021 were obtained from the Humanitarian Data Exchange open data platform (United Nations Office for the Coordination of Humanitarian Affairs, OCHA)^24^. The populations for the previous years (2015–2020) for each regional state were calculated using decreasing population growth rates provided by the EPHI as follows: Addis Ababa, 2.1%; Afar, 2.2%; Amhara, 1.7%; Benishangul Gumz, 3%; Dire Dawa, 2.5%; Gambela, 4.1%; Harari, 2.6%; Oromia, 2.9%; SNNP and Sidama 2.9%; Somali, 2.6%; and Tigray, 2.5%.

### Geographic information system (GIS) data

The original GIS file layerscorrespond to the following administrative units: regional states (13 regional states including Addis Ababa), zones (92 zones) and woredas (1040 woredas). Additionally, public domain vector map data (1:10 m scale) was retrieved from Natural Earth open-source repository and clipped to the Ethiopia national boundary (lakes, rivers, major cities and road networks)^30^.

### Epidemiological analysis

Cholera case-based and aggregated data in Microsoft Excel format were cleaned as described below and assembled after data quality verification into a single database of weekly case and death numbers per woreda using RStudio 2023.03.1^31^ with R-4.3.0 version^32^ for downstream epidemiological analyses. GIS files were managed using QGIS V3.28 Firenze^33^ and R-4.3.0^32^.

To verify the spatial data, the case locations (region, zone and woreda) were systematically verified (e.g. consistent spelling) according to the corresponding location in the GIS file attribute table. During the study period, two new regions (Sidama Region in 2020 and SWEP Region in 2021) were created within SNNP Region (GIS files, December 2021 version^34^). Cases were assigned to the new regions according to the reporting kebele localization.

In Tigray Region, to represent the most recent administrative organization, the correct woreda for each case (n = 5945) was identified based on the kebele information by overlaying the kebele-level shapefile.

Furthermore, the data for a few woredas in Amhara Region and Somali Region were merged either because they were already aggregated in the databases or because the initial localization description was ambiguous (e.g. for East Dembia and West Dembiya, many cases were listed simply as “Dembia” or “Dembiya”). For Amhara Region, (1) East Dembia and West Dembiya Woredas were merged into “Dembia (W-E)”; (2) Aykel town, Chilga 1 and Chilga 2 Woredas were merged into “Chilga (T-1–2)”; and (3) East Esite, West Esite and Mekan Eyesuse Woredas were merged into “Esite (W-E)”. For Somali Region, Degahabur Town and Degehabur were merged into “Degahabur (T-Z)” and Kebridehar Town and Kebridehar were merged into “Kebridehar (T-Z)”. In this study, the total number of health surveillance units (woreda level) is 1033.

To verify the dates of onset and admission at the health facility recorded in the line lists, the original Ethiopian dates (Ge’ez calendar) and the derived Gregorian dates were systematically verified. All records and available dates were verified (date of onset, date of admission, date of discharge and date of sampling, if any). The epi-week of onset for each case was then calculated according to the Gregorian calendar dates using the ISO week date system. If the onset date was unavailable, the date seen at the health facility was applied. The case and death observations (in the line lists and aggregated data) were aggregated by week for downstream analysis.

Duplicate case data were removed prior to analysis by identifying multiple identical entries based on the combination of the following case-based information: sex, age, patient identifier, woreda, date of onset, date seen at health facility, date of admission and status. For observations lacking the patient identifier information, duplicate lines were identified based on the following case-based information: sex, age, woreda, date of onset, date seen at health facility, date of admission and status. Observations with similar combinations of case-based information were removed.

All line lists and aggregated databases were then consolidated into a single database for further analysis. We then performed a gap analysis for the period 2016–2018 in which the total case numbers per region were verified using the regional total numbers provided by the WHO. For any data gaps identified, we requested the missing line list data. For the years 2019–2021, the total case numbers nationwide were verified using the annual totals available in the WHO Weekly Epidemiological Records. A region-level gap analysis thus could not be performed for the years 2019–2021. Epidemic curves per zone and per woreda were generated using R-4.1.1 and all times-series per woredas were verified to assess outbreak evolution over time. Any outliers and unusual backlogs were assessed with surveillance experts and corrections were applied accordingly.

### Identification and classification of cholera hotspots

Three methods were applied and compared to classify cholera hotspots in Ethiopia at the woreda level.

#### Method A

This classification method involves the analysis of three epidemiological parameters: (1) outbreak frequency, (2) outbreak duration (median, in weeks) and (3) median standardized outbreak attack rate (in 10,000 person-weeks).

To define an outbreak event in each CSU, the weekly time series were processed as follows. Sporadic cases were removed (i.e. one to two cases without reported cases during the week before and after) to mitigate potential notification biases affecting outbreak duration. The weekly number of cases were interpolated using a local polynomial fit (Package ‘interp’, function locpoly, bandwith = 0.5). The interpolation parameters were optimized to fit the outbreak period and minimum cut-off threshold defined on smoothed values to automatically extract for start and end week of each outbreak event. Observed and smoothed time-series and extracted values were manually verified for all CSUs to assess the start and end weeks of each outbreak. A minimum of ten cases was required for a transmission event to be considered an outbreak. Furthermore, two successive outbreak events separated by an inter-epidemic period ≥ six weeks were considered as two separate outbreaks.

The following epidemiological indicators were extracted for each outbreak in each CSU: the number of reported cases and deaths, outbreak start and end week, outbreak duration (in weeks), and standardized outbreak attack rate. Based on the percentile range of the three outbreak parameters (i.e. frequency, duration and standardized outbreak attack rate), each CSU was classified into four hotspot types: Type 1: area with cholera outbreaks of high frequency and extended duration, Type 2: area with cholera outbreaks of moderate frequency and extended duration, Type 3: area with cholera outbreaks of high frequency and short duration, and Type 4: area with cholera outbreaks of moderate frequency and short duration (Table 3).

**Table 3:** Summary of cholera hotspot classification methods.

Method	Description	Parameters	Classification categories
A	Three-parameter method based on outbreak characteristics	Outbreak frequency Outbreaks duration (median, in weeks) Standardized outbreak attack rate (median, in person-weeks)	Four priority categories: Type 1: Outbreak frequency ≥ 3 (P95) and median outbreak duration ≥ 10.5 weeks (P60) Type 2: Outbreak frequency = 2 (P70-P95) and median outbreak duration ≥ 10.5 weeks (P60) Type 3: Outbreak frequency ≥ 3 (P95), median outbreak duration < 10.5 weeks (P60) and median standardized outbreak attack rate ≥ 0.1 cases per 10,000 person-weeks (P60) Type 4: Outbreak frequency = 2 (P70- P95), median outbreak duration < 10.5 weeks (P60) and median standardized outbreak attack rate ≥ 0.1 cases per person-weeks (P60)
B	Two-parameter method based on yearly epidemiological indicators	Mean yearly incidence Persistence	Three priority categories: High: high mean incidence (≥ 1 case/10,000 population) and high persistence (> 5%) Medium: low mean incidence (< 1 case/10,000 population) and high persistence (> 5%) OR high mean incidence (≥ 1 case/10,000 population) and low persistence (< 5%) Low: Low mean incidence (< 1 case/10,000 population) and low persistence (< 5%)
C	Multi-parameter method based on cumulative epidemiological indicators and a cholera testing indicator (if applicable)	Cumulative incidence Cumulative mortality Persistence Cholera test positivity indicators included based on the level of representativeness: - Acceptable: positivity rate - Suboptimal: the number of years with at least one positive case - Insufficient: Test positivity indicator excluded from the calculation	Indicator scoring rules: - No cases = 0 points, - 0 and < median = 1 point, - ≥ median and < P80 = 2 points For incidence and persistence, the median and P80 were calculated for CSUs that reported at least one cholera case. For mortality, the median and P80 were calculated for CSUs that reported at least one death Priority index values (per CSU) is the sum of score for each indicator Two priority categories: Priority area above the priority index cut-off No priority area below the priority index cut-off

P60: 60th percentile, P70: 70th percentile, P80: 80th percentile P95: 95th percentile.

#### Method B

This method was used to classify CSUs based on two epidemiological indicators: (1) mean of the yearly annual incidence (per 10,000 pop.) and (2) total number of weeks with at least one reported cholera case divided by the total number of weeks in the study period (expressed as percentage)^7^. Both indicators are dichotomized into two categories (low and high); however, as no specific cut-off is proposed, the cut-off should be determined by the country authorities. For this analysis, the thresholds applied were defined in the Ethiopian National Cholera Plan as follows: “high incidence” corresponds to values ≥ 10 cases per 100,000 population and “high persistence” corresponds to values ≥ 5% (Table 3). The CSUs were then classified into three priority levels: (1) high (areas with high incidence and high persistence), (2) medium (areas with low incidence and high persistence, or with high incidence and low persistence) and (3) low (areas with low incidence and low persistence).

#### Method C

This method was developed to rank priority areas for cholera prevention and control interventions in countries with high to moderate cholera transmission based on retrospective data collected over the recent five to 15 years^13^. Indicators used to calculate the priority index were derived from the weekly number of cases for each CSU over the course of the study period as follows: (1) cumulative incidence (cumulative number of cholera cases reported per 10,000 person-years), (2) cumulative mortality (cumulative number of cholera-related deaths reported per 10,000 person-years), (3) persistence (percentage of weeks with at least one reported suspected cholera case over the total number of weeks of the study period). A fourth indicator, cholera test positivity, can be considered according to the representativeness of cholera testing among suspected cases, which is determined using the weekly testing coverage (percentage of weeks with at least one suspected case tested for cholera (regardless of the testing method) among weeks with at least one suspected case reported). If the level of representativeness is acceptable, the cholera test positivity indicator selected is the overall positivity rate (percentage). If the level of representativeness is considered suboptimal, the number of years with at least one case tested positive for cholera (regardless of the testing method) is instead included as a test indicator. If the level representativeness is considered insufficient, the cholera test indicator is not included. To apply Method C, we assessed whether the cholera test coverage indicator could be included in the analysis. Over the course of the study period, 221 CSUs (42% of the total) performed cholera testing of one or more suspected case(s) in at least one week, which indicates that the cholera test representativeness is insufficient. As a result, the priority index for this database was based solely on the three epidemiological indicators (incidence, mortality, and persistence).

The values for incidence, mortality and persistence were then converted into separate scores according to a four-point scale based on the 50th and 80th percentiles of distribution (Table 3). The final priority index was calculated taking the sum of the scores for each indicator. The initial list of priority areas was defined using the median value of the priority score.

### Cartography

All maps were generated using the GIS files described above and the software QGIS V3.28 Firenze^33^ and R-4.3.0^32^ (with ggmap package).

### Ethical considerations

The research protocol was submitted for ethics approval by the EPHI Institutional Review Board in March 2021. The research protocol was approved by the EPHI Institutional Review Board on June 14, 2021. The EPHI Institutional Review Board waived the informed consent requirement. All experiments were performed in accordance with the relevant guidelines and regulations.

### Supplementary Information

Supplementary Information.

## Data Availability

Data are available from the authors upon reasonable request and with the permission from Yeshambel Worku (workuyeshambel19@gmail.com) and Moti Edosa (motiedosa7@gmail.com).

## Abbreviations

AWD: Acute watery diarrhea
CSU: Cholera surveillance unit
EPHI: Ethiopian Public Health Institute
GTFCC: Global task force on cholera control
SNNP: Southern nations, nationalities, and peoples
SWEP: South West Ethiopia Peoples
UNICEF: United Nations Children’s Fund
WASH: Water, sanitation and hygiene
WCARO: West and Central Africa Regional Office
WHO: World Health Organization
