# Supplementary material for: Comparison of analysis methods to classify cholera hotspots in Ethiopia from 2015 to 2021
Source: Sci Rep. 2024 Apr 3;14:7377. doi: 10.1038/s41598-024-56299-5 (PMC10991413; doi:10.1038/s41598-024-56299-5)
Supplement: Supplementary file 1 — Supplementary Information. [file 41598_2024_56299_MOESM1_ESM.docx]

## Supplementary material

Supplementary material 1: Maps of the epidemiological indicators applied in Method A


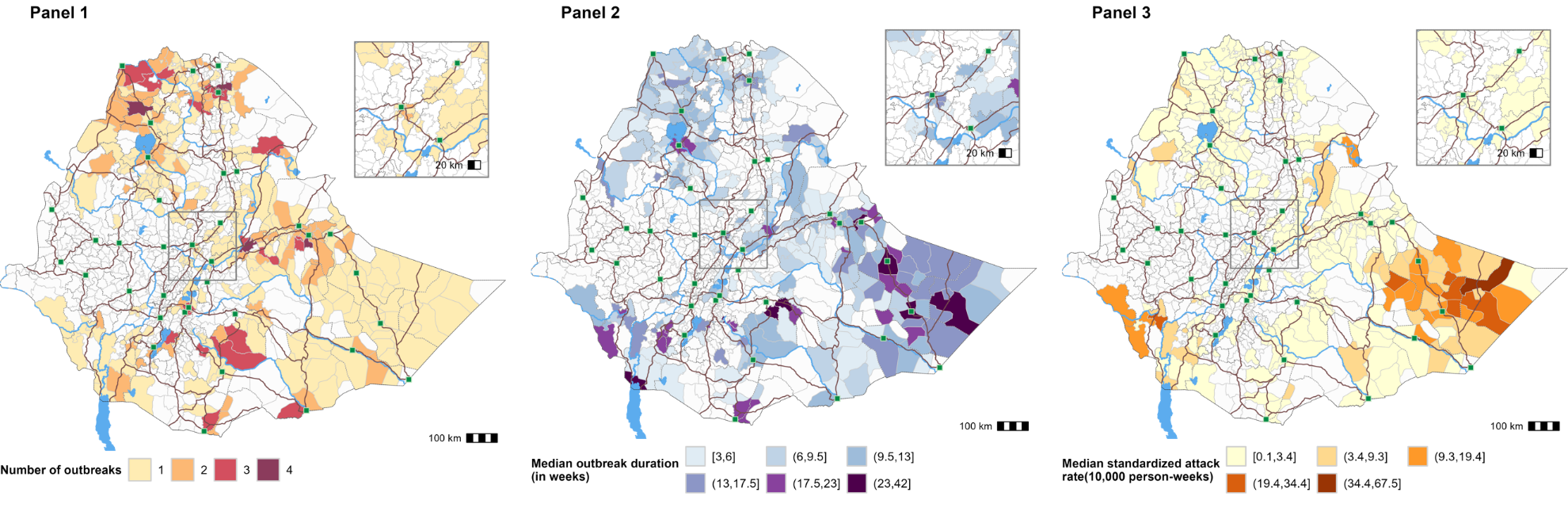


Legend: Dark brown lines correspond to roads, green squares correspond to main urban centers, and blue lines and areas correspond to waterbodies. The maps were generated using the software QGIS V3.28 Firenze and R-4.3.0 (with ggmap package).

Supplementary material 2: Maps of epidemiological indicators applied in Method B


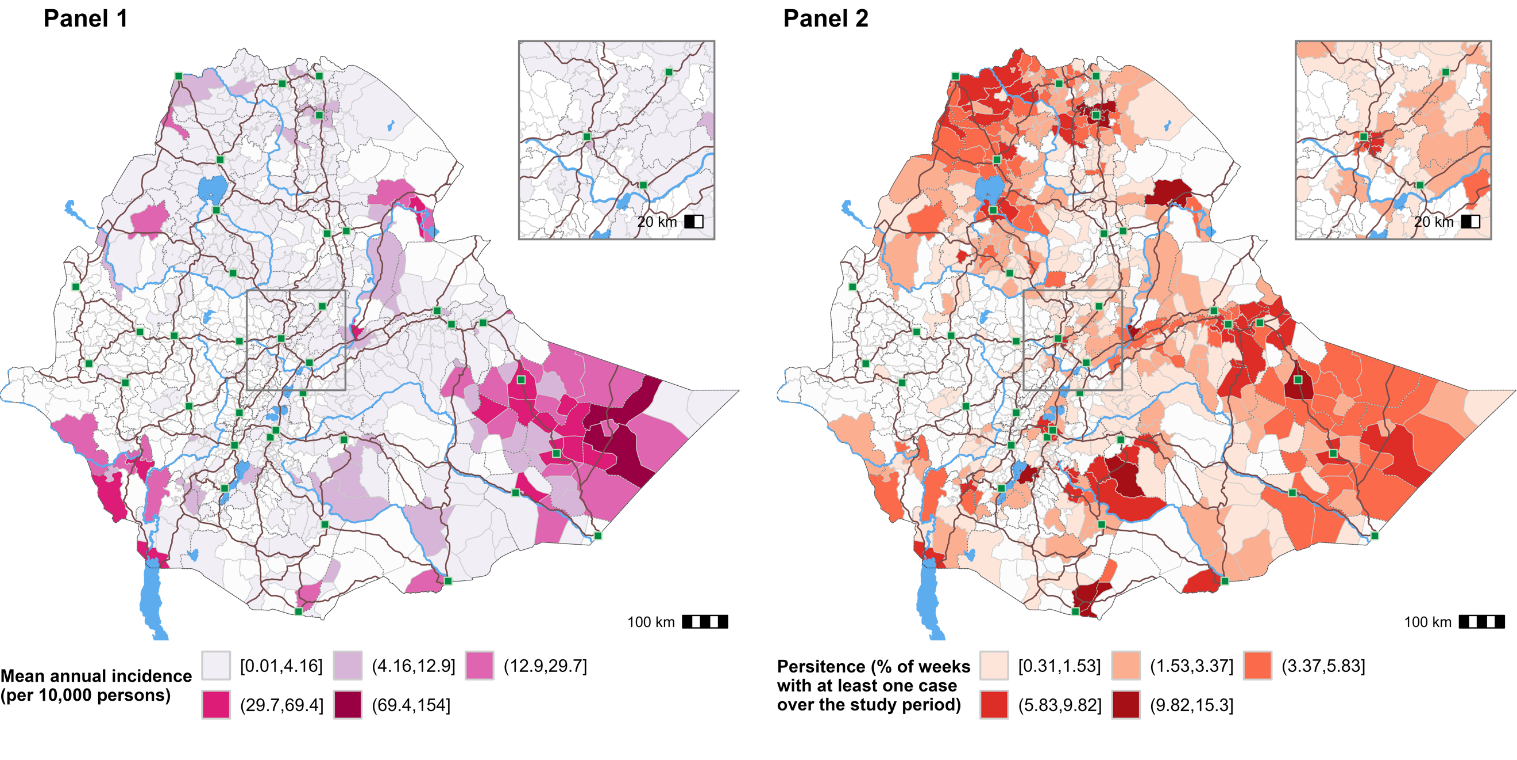


Legend: Dark brown lines correspond to roads, green squares correspond to main urban centers, and blue lines and areas correspond to waterbodies. The maps were generated using the software QGIS V3.28 Firenze and R-4.3.0 (with ggmap package).

Supplementary material 3: Maps of epidemiological indicators applied in Method C


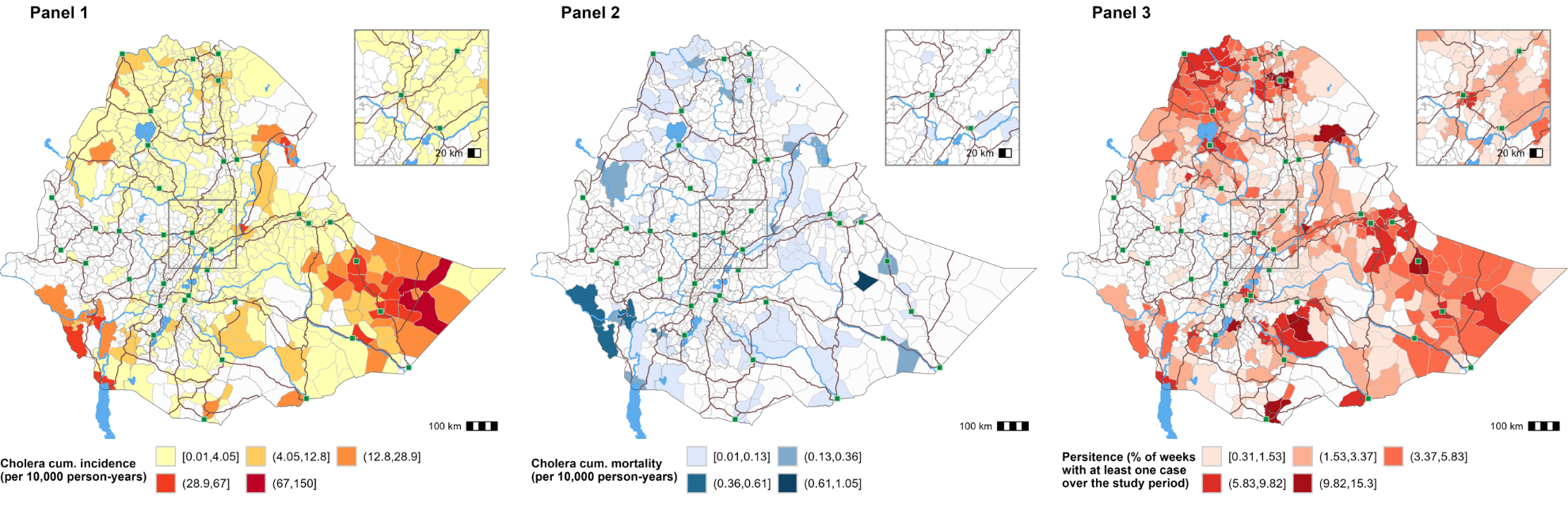


Legend: Dark brown lines correspond to roads, green squares correspond to main urban centers, and blue lines and areas correspond to waterbodies. The maps were generated using the software QGIS V3.28 Firenze and R-4.3.0 (with ggmap package).
